# Ommaya reservoir converted to permanent cerebrospinal fluid shunt in neonatal posthemorrhagic hydrocephalus: a risk factors analysis

**DOI:** 10.3389/fsurg.2026.1800808

**Published:** 2026-06-02

**Authors:** Cao Xuehui, Feng Feng, Li Xin

**Affiliations:** 1 Pediatric Neurosurgery Department, Hebei Children’s Hospital, Shijiazhuang, Hebei, China; 2 Hepatobiliary and Pancreatic Surgery Department, the First Hospital of Hebei Medical University, Shijiazhuang, Hebei, China

**Keywords:** apgar score, cerebrospinal fluid protein, neonatal posthemorrhagic hydrocephalus, ommaya reservoir, ventriculoperitoneal shunt

## Abstract

**Objective:**

To identify factors influencing the transition to a permanent ventriculoperitoneal (VP) shunt in neonates with severe intraventricular hemorrhage (IVH) initially managed with an Ommaya reservoir.

**Methods:**

This retrospective study involved 26 neonates diagnosed with IVH in Hebei Children's Hospital from January 2020 to January 2025. These patients were diagnosed with Papile grade III or IV IVH and treated with an Ommaya reservoir. They were categorized into two groups: a permanent shunt group (*n* = 10) and a non-permanent shunt group (*n* = 16). These two groups were compared based on demographic, clinical, and cerebrospinal fluid (CSF) variables.

**Results:**

Of all study subjects, 10 patients (38.5%) received a permanent VP shunt placement. The 5-minute Apgar score was significantly higher in the permanent shunt group (9.40 ± 1.08) compared to that of the control group (7.25 ± 2.50, *p* = 0.021). Also, the CSF protein levels were notably increased in the permanent shunt group (2.80 ± 1.43 g/L vs. 1.81 ± 0.79 g/L, *p* = 0.028). And, ROC curve analysis revealed that a 5-minute Apgar score exceeding 8.5 was predictive of the requirement for permanent cerebrospinal fluid shunting, with corresponding sensitivity and specificity values of 80.0% and 68.7%, respectively. Moreover, multivariate analysis indicated that a 5-minute Apgar score greater than 8.5 remained an independent predictor of permanent shunt placement (adjusted OR 12.24, 95% CI 1.11–134.75, *p* = 0.041). In contrast, CSF protein ≥2.04 g/L (adjusted OR 4.82, 95% CI 0.61–38.41, *p* = 0.138) and sex did not show statistical significance.

**Conclusion:**

In this retrospective study, a higher 5-minute Apgar score (>8.5) was identified as an independent predictor for the requirement of a permanent VP shunt after Ommaya reservoir placement in neonates with severe IVH. This seemingly counterintuitive result may be explained by the impact of a more robust neonatal circulation on the initial hemorrhage volume. Although elevated CSF protein was associated with shunt dependency, it was not an independent predictor. These findings underscore the intricate pathophysiological mechanisms of posthemorrhagic hydrocephalus.

## Introduction

Posthemorrhagic hydrocephalus remains a significant challenge in the field of neonatal neurocritical care. The pathological mechanism centers on germinal matrix hemorrhage, where the immaturity and fragility of cerebral blood vessels increase their susceptibility to fluctuations in cerebral blood flow, ultimately triggering intraventricular hemorrhage (1, 2). After IVH, approximately 25%–50% of high-grade (Papile grade III/IV) cases will progress to posthemorrhagic hydrocephalus (PHH), requiring cerebrospinal fluid diversion therapy (3).

VP shunt implantation is a definitive treatment modality for patients with PHH (4). However, due to the high risks of infection and malfunction associated with this procedure, as well as the technical difficulties in treating low-weight neonates, immediate application is often not feasible (5, 6). Therefore, temporary neurosurgical procedures (TNPs), such as external ventricular drainage, ventriculosubgaleal shunting, and the implantation of subcutaneous ventricular access devices (e.g., Ommaya reservoirs), serve as important interim measures (7). These procedures help control the progressive enlargement of the ventricles until the infant is in a suitable condition for permanent shunt placement (5, 8).

Although these temporary neurosurgical procedures are effective in managing acute hydrocephalus, a considerable number of infants eventually require conversion to a VP shunt (9). Current research has extensively compared the failure and infection rates among different temporary neurosurgical procedures (10, 11). However, there is still insufficient exploration into reliable predictive factors for shunt dependency in the specific population treated with Ommaya reservoirs. Identifying these factors is crucial for prognosis assessment, family counseling, and potentially optimizing the timing of definitive intervention.

Therefore, we conducted a retrospective analysis of neonates with severe IVH who were treated with Ommaya reservoirs. The study aimed to identify demographic, clinical, and CSF biochemical factors during the perioperative period linked to a higher risk of permanent VP shunt placement.

## Methods

### Study population

A retrospective observational study was conducted on all patients born between January 2020 and January 2025 and treated at Hebei Children's Hospital. Inclusion criteria included: (1) Papile grade III or IV IVH; (2) consent for Ommaya reservoir placement; (3) absence of infections in the subdural space; (4) progressive hydrocephalus with a ventricular index exceeding the 97th percentile by 4 mm.

From January 2020 to January 2025, 31 patients received Ommaya reservoir placement. One case was excluded due to asphyxia, two due to infection, and two due to withdrawal of care by family members. Asphyxia and care withdrawal were not associated with the primary outcome of shunt placement. In contrast, the exclusion of the two infected cases was highly correlated with the study outcome. Infection (ventriculitis) is not merely a random complication, but a biologically relevant factor that can directly exacerbate adverse outcomes in the late stage. Infection induces aggravated intraventricular inflammation, protein extravasation, and potential septation formation—all well-recognized factors that impede hematoma resolution and accelerate hydrocephalus progression. Finally, the study included 26 patients, with 10 receiving permanent ventriculoperitoneal shunts (shunt group) and 16 not requiring them (non-permanent shunt group).

### Variable measurement

This study recorded and analyzed the demographic data, clinical variables, and the complication rates associated with permanent shunt placement. Patients received transcranial sonography and CT/MRI both pre- and post-Ommaya reservoir implantation, with ventricular index changes recorded following reservoir tapping.

Lumbar puncture was performed based on a standardized clinical protocol. Specifically, when the ventricular index exceeded the 97th percentile and tended to increase to the 97th percentile +4 mm (serving as the reference threshold for initiating PHVD/PHH intervention), lumbar puncture for cerebrospinal fluid drainage was conducted if the infant's vital signs were unstable and could not tolerate surgery. In contrast, for stable patients who met the same ventricular index criteria, Ommaya reservoir implantation was preferred. The standardized criteria for cerebrospinal fluid drainage via Ommaya reservoir tapping were defined as follows: daily tapping of the Ommaya reservoir was performed for one month, with intermittent cranial ultrasound and MRI re-evaluations conducted during this period. After one month, the tapping frequency was adjusted to once every 3 days, accompanied by daily head circumference monitoring. A repeat cranial MRI was performed 15 days later: if no ventricular dilation was observed, reservoir tapping was discontinued; if ventricular dilation persisted, tapping was continued until the patient met the criteria for permanent shunt placement, which included a body weight of more than 2000g, a CSF protein level of less than 1 g/L, and stable clinical status.

Subsequently, CSF extraction commenced within 24 h post-surgery, with 5–15 mL/kg removed daily for two weeks. The drainage volume was adjusted based on clinical manifestations and ultrasound findings. After 2 weeks, CSF was extracted every 48–72 h until the tapping was gradually stopped. Nevertheless, if there was clinical deterioration or poor ultrasound response, daily tapping was performed. Tapping was continued for 1–2 months until the patient's weight reached 2000g and the CSF protein count was <1 g/L, after which the tapping was gradually discontinued. A ventriculoperitoneal shunt was implanted if the patient exhibited progressive ventricular widening.

Moreover, CSF variables measured included total protein, glucose, lactate, white blood cells, and red blood cells. In this study, all CSF specimens were collected concurrently with Ommaya reservoir implantation surgery. All variables were recorded at the time of Ommaya reservoir implantation and each lumbar puncture. In cases initially treated by internists, lumbar puncture was performed as a temporary measure before Ommaya reservoir placement. For the final analysis, the mean values of CSF variables were calculated. A permanent shunt was placed when the patient weighed over 2,000g, had a CSF protein count below 1 g/L, was clinically stable, and exhibited a progressively increasing ventricular index following the discontinuation of Ommaya reservoir tapping.

### Statistical analysis

Categorical data were described as counts and percentages, whereas normally distributed continuous variables were expressed as means and standard deviations, and non-normally distributed ones were presented as medians and interquartile ranges. For bivariate analysis, Fisher's exact test was used to evaluate categorical outcomes, and continuous data were assessed via the Wilcoxon rank-sum test. The log-rank test was employed to compare the time until ventriculoperitoneal shunt implantation. ROC curve analysis was conducted to assess the sensitivity and specificity across various CSF protein thresholds, selecting the cutoff that maximized classification accuracy. All analyses included reported *P* values. The analysis was conducted using R 4.4.2 software.

## Results

A total of 10 patients (38.46%) underwent ventriculoperitoneal shunt implantation The median gestational age of the study cohort was 35.50 weeks (range: 28.60–39.10), and the median birth weight reached 2,301.00 g, with individual values varying from 1,270.00 g to 3,395.00 g. Demographic and clinical variables showed no significant differences between the groups (Tables 1–3).

**Table 1:** Baseline demographic data and clinical indicators.

**Variable**	**Shunt group**	**Nonpermanent shunt group**	***P* Value**
No. Of patients	10	16	
Male, %	60 (6)	75 (12)	0.66
Vaginal delivery	60 (6)	31 (5)	0.23
Gestational age, wks	34.97 ± 5.79	33.61 ± 4.82	0.65
Age of onset, days	13.60 ± 10.12	13.06 ± 10.34	0.94
Reservoir time, days	22.60 ± 15.03	21.00 ± 16.60	0.9
Birth weight, g	2,656.50 ± 1,209.36	2,117.00 ± 963.01	0.37
VI index, mm Left	2.62 ± 0.48	2.34 ± 0.32	0.14
Right	2.28 ± 0.44	2.07 ± 0.31	0.15
Papile grade III	3	7	
IV	7	9	0.68
Lumbar taps, n(%)	8	14	0.63

**Table 2:** CSF analysis data.

**Variable**	**shunt group**	**nonpermanent shunt group**	***P* Value**
Glucose (mmol/L)	0.92 ± 0.64	1.07 ± 0.82	0.541
Protein (g/l)	2.80 ± 1.43	1.81 ± 0.79	0.028
Lactate (mmol/l)	3.20 ± 0.94	2.46 ± 0.73	0.332
LDH (U/l)	280 (208, 682)	339 (206, 796)	0.417
RBC (*10^9^)	1.88 ± 0.86	1.07 ± 0.87	0.748
WBC (*10^6^)	78.1 ± 63.34	55.06 ± 38.67	0.221
CSF extraction >10 mL/kg/day	6 (60%)	8 (50%)	0.701

LDH, lactate dehydrogenase.

**Table 3:** Logistic regression analysis with multiple covariates for predicting permanent shunt placement.

**Indicator**	**Unadjusted OR**	***P* Value**	**Adjusted OR**	***P* Value**
Sex	0.5 (0.09–2.73)	0.352	0.15 (0.01–1.96)	0.146
5-minute Apgar score	8.8 (1.35–57.43)	0.021	12.24 (1.11–134.75)	0.041
Protein ≥2.04 g/L	5.13 (9.22–28.57)	0.063	4.82 (0.61–38.41)	0.138

### Clinical manifestations and comorbidities

**Figure 1:**
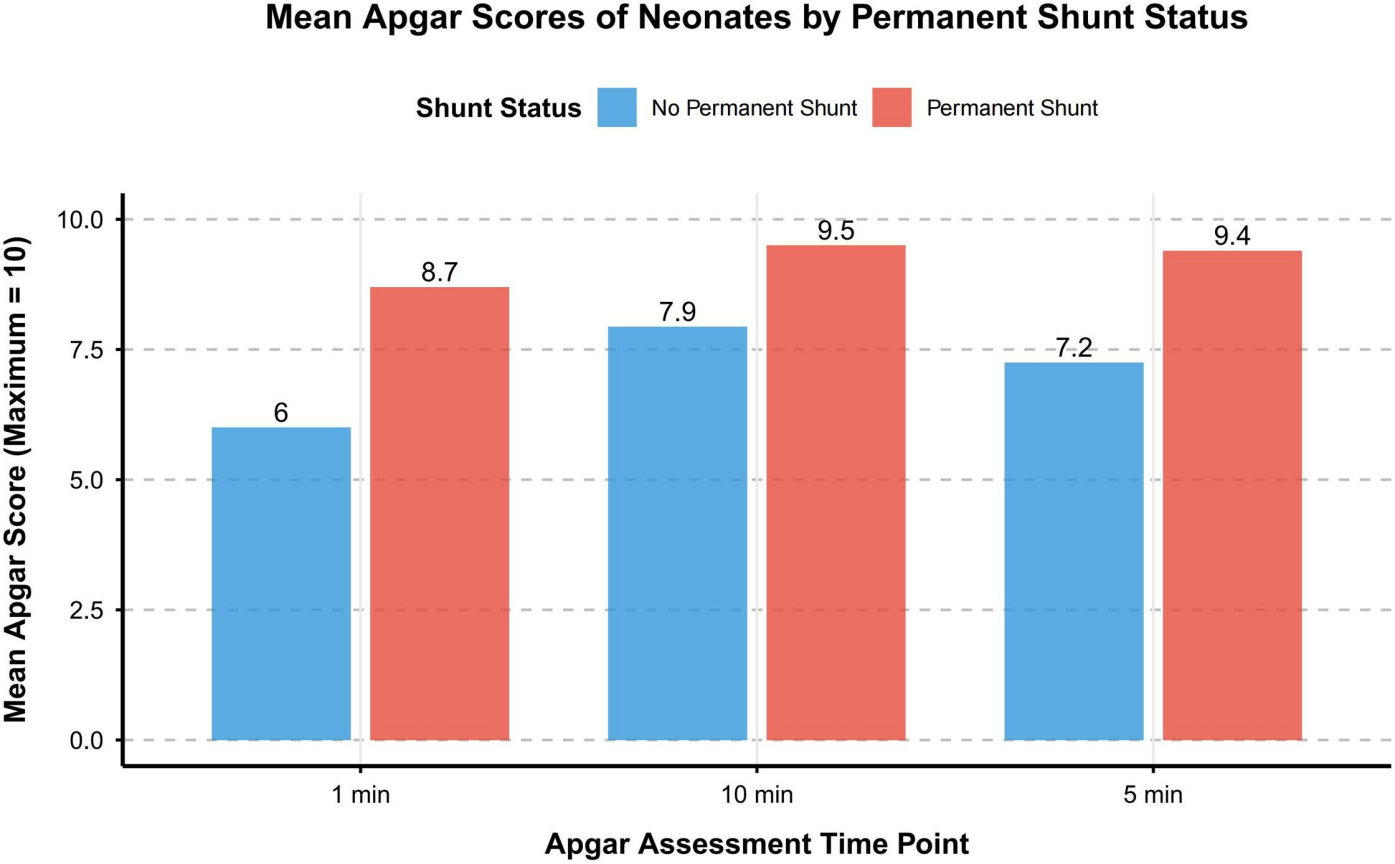
Mean apgar scores of neonates by permanent shunt Status.

**Figure 2:**
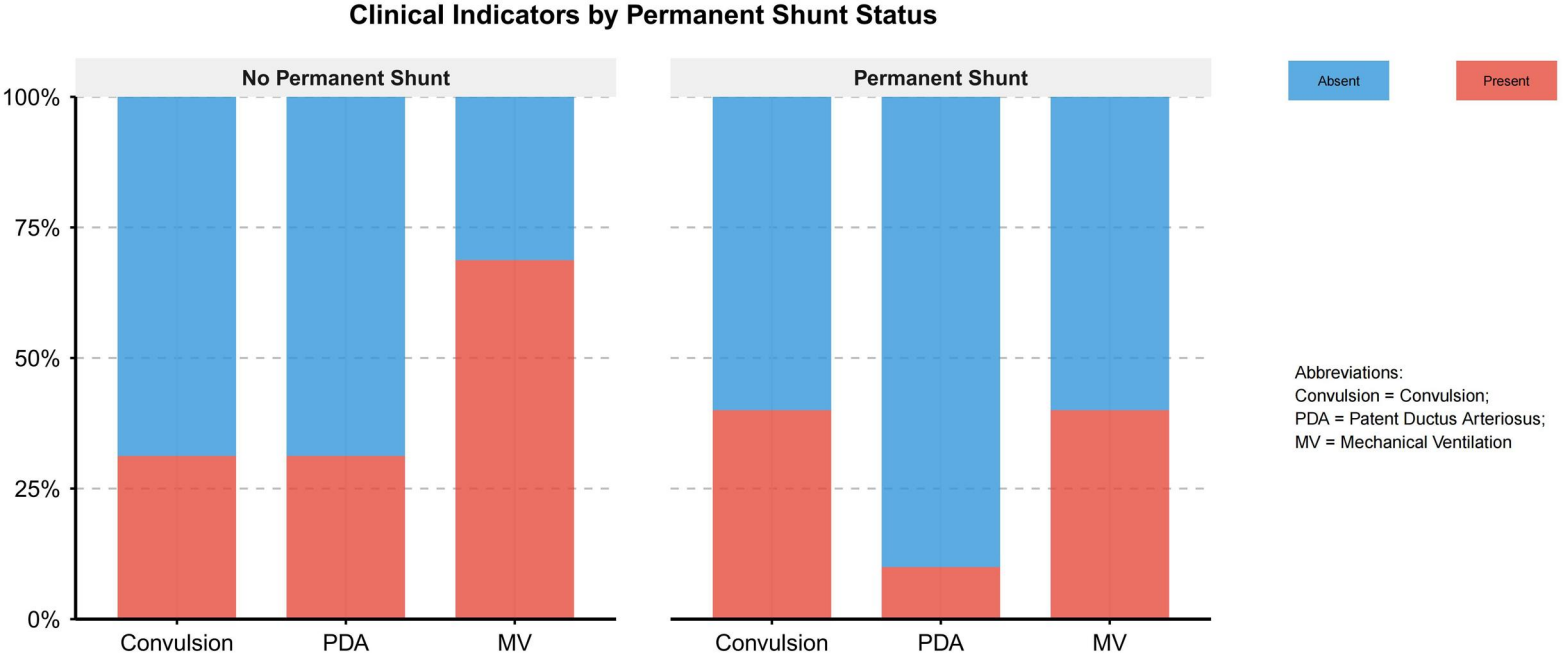
Clinical indicators by permanent shunt Status.

Clinical manifestations and comorbidities recorded included Apgar scores, seizures, invasive mechanical ventilation, and patent ductus arteriosus (PDA). The permanent shunt group exhibited a significantly higher 5-minute Apgar score (9.40 ± 1.08) compared to the non-permanent shunt group (7.25 ± 2.50) (*p* = 0.021).2) (Figure 1). The 1-minute Apgar score showed a non-significant trend toward higher values in the permanent shunt group (8.70 ± 2.11) in comparison with the non-permanent group (6.00 ± 3.01) (*p* = 0.089). The 10-minute Apgar scores exhibited no statistically significant intergroup differences (*p* = 0.16). The incidence of seizures, invasive mechanical ventilation, and PDA was similar in both groups (Figure 2).

There was no statistically significant correlation between hemorrhage grade, mode of delivery, and 5-minute Apgar score. Among the 26 study subjects, 13 had a 5-minute Apgar score >8.5, and 13 had a score ≤8.5. In terms of mode of delivery, among 11 vaginal deliveries, 7 cases (63.6%) had an Apgar score >8.5, and 4 cases (36.4%) had a score ≤8.5, while among 15 cesarean sections, 6 cases (40.0%) had an Apgar score >8.5, and 9 cases (60.0%) had a score ≤8.5, with no statistically significant correlation between mode of delivery and 5-minute Apgar score (*P* = 0.428). For hemorrhage grade, among 10 cases of grade III hemorrhage, 5 cases (50.0%) had an Apgar score >8.5, and 5 cases (50.0%) had a score ≤8.5, and among 16 cases of grade IV hemorrhage, 8 cases (50.0%) had an Apgar score >8.5, and 8 cases (50.0%) had a score ≤8.5, with no statistically significant correlation between hemorrhage grade and 5-minute Apgar score.

### ROC curve analysis for 5-minute apgar score

**Figure 3:**
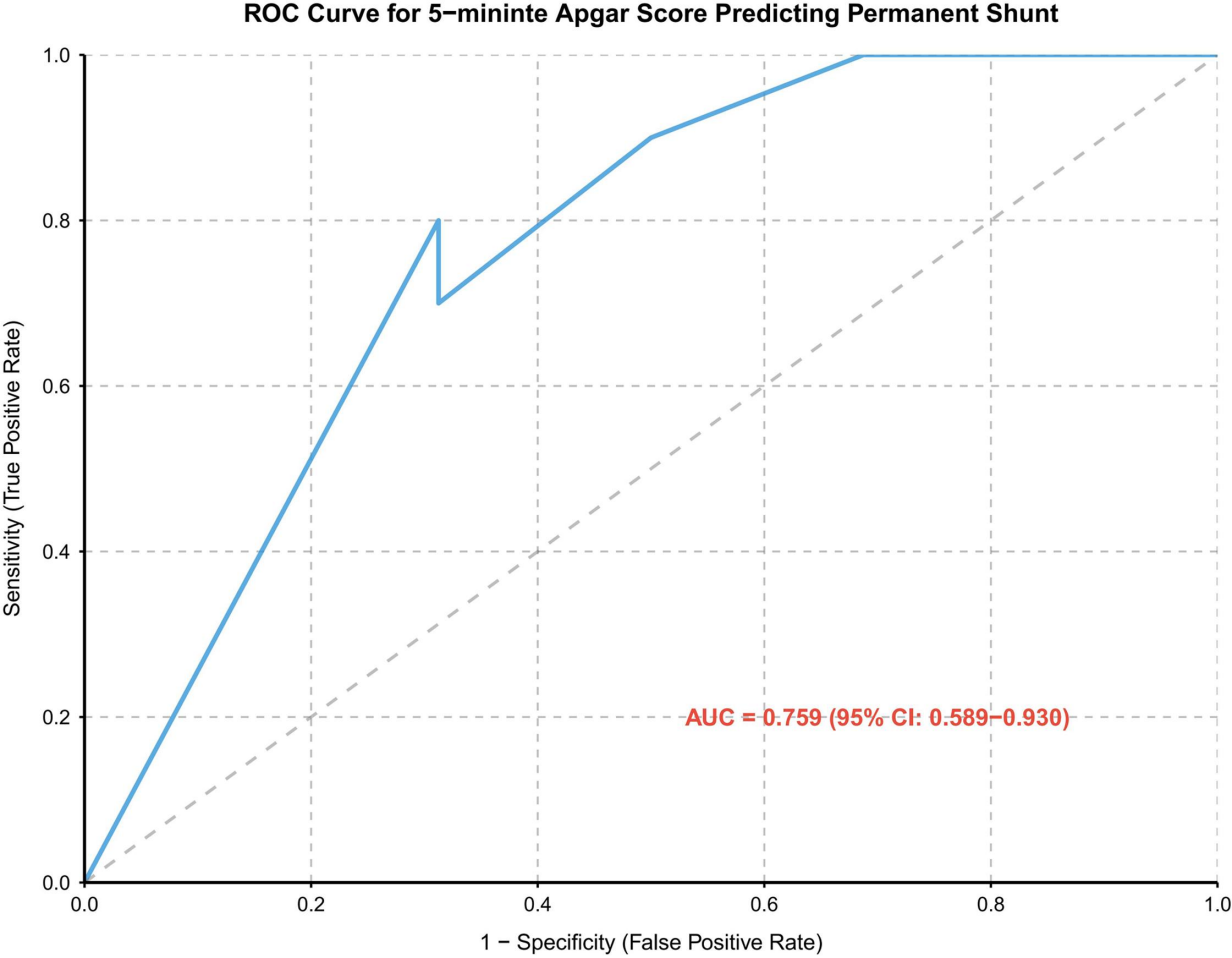
ROC Curve 5-minutes Apgar score predicting Permanent Shunt

ROC curve analysis was implemented to ascertain the cutoff value for the 5-minute Apgar score (Figure 3). The 5-minute Apgar score demonstrated the highest area under the curve (AUC = 0.759, 95% CI 0.589–0.930). Using a cutoff value of 8.5, the sensitivity was 80%, the specificity was 68.7%, and the positive likelihood ratio was 2.56.

### CSF variables

A higher protein count prior to Ommaya reservoir placement was significantly linked to the necessity for permanent shunt placement. The permanent shunt group exhibited elevated CSF lactate and white blood cell levels; however, these differences lacked statistical significance. The two study groups did not exhibit any significant variations in glucose, lactate dehydrogenase, or RBC counts. The necessity for extracting greater than 10 mL/kg/day of CSF during Ommaya reservoir use was not significantly linked to the requirement for a permanent shunt.

### Multivariate logistic regression analysis

Logistic regression analysis with multiple covariates was performed including sex, 5-minute Apgar score, and protein level. The study found that the 5-minute Apgar score was the sole independent predictor of permanent shunt placement, presenting an unadjusted odds ratio (OR) of 8.8 (95% CI: 1.35–57.43, *p* = 0.021) as well as an adjusted OR of 12.243 (95% CI: 1.11–134.75, *p* = 0.041). The analysis excluded additional variables to prevent model instability caused by the small sample size.

## Discussion

Managing posthemorrhagic hydrocephalus in newborns presents a significant challenge for both neonatologists and neurosurgeons. As early as the 1980s, temporary placement of a subcutaneous ventricular catheter reservoir was proposed as a simple, safe, and effective method to prevent progressive ventricular enlargement (8, 12). Studies have shown that initial treatment with a permanent shunt is associated with poorer cognitive outcomes compared to temporary intervention. Therefore, early temporary treatment is recommended, as it may allow some patients to wean off treatment and optimize the timing of permanent shunt placement by avoiding treatment delays (13).

This retrospective study examined factors influencing the rate of conversion to a permanent shunt. In our institutional series of 26 neonates who underwent Ommaya reservoir placement, 10 (38.5%) subsequently required conversion to a permanent shunt. Multivariate logistic regression analysis determined that a 5-minute Apgar score greater than 8.5 independently predicts the risk of permanent shunt conversion. Additionally, a CSF protein level exceeding 2.04 g/L was significantly associated with permanent shunt placement. Most previous studies have focused on comparing the rate of conversion to permanent shunt among different temporary measures, while research on the factors influencing conversion rates is relatively limited (10, 11). A multicenter study identified a fully bulging fontanelle and increased ventricular size as the sole independent predictors for permanent shunt placement, among various clinical variables like bradycardia, apnea, widened cranial sutures, fronto-occipital ratio, clot radiodensity, and clot anatomical location (14). A retrospective multivariate regression analysis identified elevated CSF lactate levels as an independent risk factor for shunt dependency (15).

Our observation concerning CSF protein aligns with a mounting body of research that points to hemorrhagic CSF components as key factors in the development of PHH (7). Studies on posthemorrhagic hydrocephalus pathogenesis primarily examine hemorrhagic CSF components including erythrocytes, hemoglobin, iron elements, thrombin, and subarachnoid fibrosis. Hematoma-induced mass effect, together with concurrent ventricular inflammation, is considered the main mechanism involved in the development of PHH (16, 17). After intraventricular hemorrhage, a subsequent increase in erythrocytes in the hemorrhagic CSF is rare; instead, these cells undergo gradual lysis. Elevated total CSF protein (CSF-TP) primarily results from erythrocyte destruction, platelet degranulation and leukocyte secretion, and the discharge of materials from damaged brain parenchyma (18, 19). Research indicates a significant association between PHH in preterm infants and biomarkers like CSF amyloid, Cell adhesion molecule L1, neural cell adhesion molecule-1, along with other neurodevelopmental proteins (20).

Clinically, increased concentrations of proteins including TGF-*β*1, thrombopoietin, carbonic anhydrase-I, peroxiredoxin-2, S-100 protein, glial fibrillary acidic protein, neuron-specific enolase (NSE), and vascular endothelial growth factor have been observed in the cerebrospinal fluid of hydrocephalus patients (6). The hypothesis suggests that excessive protein buildup in the ventricular system impedes CSF absorption, contributing to hydrocephalus development. A longitudinal study on pediatric PHH found that changes in CSF transferrin (increased) and ferritin (decreased) levels were associated with variations in ventricular size (21). Proteomic analysis of CSF in PHH cases reveals significantly increased levels of interleukin-10, interleukin-6, interleukin-8, matrix metalloproteinase-7, and matrix metalloproteinase-9 (22).

Patients diagnosed with chronic post-traumatic hydrocephalus requiring shunt dependence exhibited significantly elevated CSF levels of Type I procollagen C-terminal propeptide, type III procollagen N-terminal propeptide, hyaluronan, and laminin compared to control subjects. The findings indicate substantial fibrosis in the subarachnoid cavity and arachnoid granulation structures, potentially underlying chronic hydrocephalus. The proteomic profile of hemorrhagic CSF is highly complex, and many of these proteins may contribute to complications associated with PHH (23). In summary, accumulating evidence highlights a close relationship between CSF proteins and the pathophysiology of PHH. Despite losing independence in our multivariate model, likely due to the modest sample size and correlation with other factors, the protein level's strong signal in univariate analysis underscores its pathophysiological significance.

While CSF protein levels showed a statistically significant association with the need for permanent shunt placement in univariate analysis, this association did not retain statistical significance in the subsequent multivariate logistic regression model. This suggests that CSF protein levels are not an independent risk factor for shunt dependency. Instead, their predictive value is likely confounded by multiple interrelated clinical variables, including the initial volume of intraventricular hemorrhage, the timing of hemorrhage onset, the infant's gestational age and postnatal developmental stage, as well as the integrity of the infant's cerebrospinal fluid circulation dynamics.

The key finding of our multivariate analysis is that a higher 5-minute Apgar score (specifically >8.5) emerged as an independent predictor for permanent shunt placement, with an adjusted odds ratio of 12.24. At first glance, the association between a higher 5-minute Apgar score and an increased risk of shunt placement seems counterintuitive, as a lower Apgar score is typically indicative of perinatal depression and poorer overall outcomes. One plausible explanation for this finding lies in the unique pathophysiology of posthemorrhagic hydrocephalus. The 5-minute Apgar score assesses a newborn's cardiorespiratory and neurological condition at birth, indicating their initial adaptation to life outside the womb. A higher score may indicate a more robust systemic circulation and, consequently, a greater ability to maintain cerebral perfusion pressure in the immediate postnatal period. In the context of a fragile germinal matrix, this sustained or potentially fluctuating higher cerebral blood flow may predispose to a larger initial IVH or prolonged bleeding, leading to a greater intraventricular clot burden (16). A larger clot volume is a well-known risk factor for more severe ventriculomegaly and impaired CSF absorption, due to the mass effect and the subsequent intense inflammatory and fibrotic responses it induces (24, 25). Therefore, infants with higher Apgar scores may have experienced a more severe hemorrhagic insult, increasing the likelihood of progressive, shunt-dependent hydrocephalus (25). Furthermore, the significantly higher CSF protein levels in the shunt group (2.80 g/L vs. 1.81 g/L, *p* = 0.028) are consistent with this concept, as elevated protein levels are a direct result of erythrocyte lysis and the subsequent inflammatory cascade within the ventricles. High protein levels may obstruct CSF reabsorption at the arachnoid granulations (26).

### Study limitations and implications

When interpreting the results, several important limitations in our study merit consideration. First, the retrospective design inherently carries the risk of selection bias and information bias. The management decisions, including the timing of Ommaya reservoir placement and the final decision to perform a shunt, were made based on clinical judgment rather than a standardized protocol, which may have introduced confounding factors. Second, the small sample size (*n* = 26) is a critical limitation. This significantly reduces the analysis's statistical power, heightening the likelihood of Type II errors, such as missing true associations like those involving CSF protein in the multivariate model. The limited number of events (10 shunt placements) renders the multivariate logistic regression model unstable, as indicated by the broad confidence interval for the adjusted odds ratio of the 5-minute Apgar score (1.11–134.75), thereby constraining the precision of our risk estimate. As a result, we were unable to include all potentially relevant variables in the model. Third, being a single-center study, our results may not be applicable to other institutions with varying patient demographics or clinical management practices. And, the study population was limited to late preterm infants; thus, the generalization of our findings to other preterm infant subgroups should be made with caution. Finally, the lack of volumetric analysis of the initial IVH clot or serial ventricular volumes represents an unmeasured confounding factor that could have provided a more direct link between hemorrhage severity and the observed outcomes.

### Future directions

The unexpected association between a higher 5-minute Apgar score and shunt dependency warrants further investigation in larger, prospective, multicenter studies. Such studies should aim to standardize treatment protocols and systematically collect data on potential confounding factors, including quantitative measurements of initial IVH volume via MRI and serial ventricular size measurements. Future research should also incorporate longitudinal CSF sampling for proteomic and inflammatory biomarker analysis to better understand the temporal dynamics of the pathways leading to fibrosis and hydrocephalus. This will help clarify whether the Apgar score is merely a surrogate for hemorrhage volume or is associated with a distinct biological phenotype of PHH. Ultimately, validating a predictive model that integrates clinical factors (such as the Apgar score) with neuroimaging and CSF biomarkers could enable early identification of neonates at the highest risk of shunt dependency, facilitating targeted interventions or enrollment in novel clinical trials aimed at preventing progressive hydrocephalus.

## Conclusion

In this retrospective study of neonates with severe IVH treated using Ommaya reservoirs, a 5-minute Apgar score exceeding 8.5 was independently linked to a higher likelihood of needing a permanent ventriculoperitoneal shunt. This finding may reflect the impact of a more robust neonatal circulation on the severity of the initial intraventricular hemorrhage. Although elevated CSF protein was significantly associated with shunt placement in univariate analysis, it was not an independent predictor in our model. These results highlight the complex interaction between systemic physiology at birth and the local pathophysiology of posthemorrhagic hydrocephalus. The study's small sample size and retrospective design necessitate cautious interpretation of the results, highlighting the need for validation in larger prospective cohorts.

## Data Availability

The original contributions presented in the study are included in the article/Supplementary Material, further inquiries can be directed to the corresponding author.
